# 减低强度预处理并更换供者二次异基因造血干细胞移植治疗移植后复发恶性血液病44例临床研究

**DOI:** 10.3760/cma.j.issn.0253-2727.2023.06.004

**Published:** 2023-06

**Authors:** 永强 赵, 艳智 宋, 智慧 李, 帆 杨, 腾 徐, 飞飞 李, 冬芳 杨, 彤 吴

**Affiliations:** 北京高博博仁医院造血干细胞移植科，北京 100070 Department of Bone Marrow Transplantation, Beijing Gobroad Boren Hospital, Beijing 100070, China

**Keywords:** 二次造血干细胞移植, 减低强度预处理, 恶性血液病, 供者, Second allogeneic hematopoietic stem cell transplantation, Reduced-intensity conditioning, Hematological malignancy, Donor

## Abstract

**目的:**

评估减低强度预处理（RIC）、更换供者二次异基因造血干细胞移植（二次移植）治疗移植后复发恶性血液病的疗效及安全性。

**方法:**

纳入2018年4月至2021年6月于北京高博博仁医院造血干细胞移植科接受RIC、更换供者二次移植的44例移植后复发恶性血液病患者，回顾性分析其临床资料。

**结果:**

①全部44例患者中男21例，女23例，中位年龄25（7～55）岁；急性B淋巴细胞白血病23例，急性T淋巴细胞白血病/T淋巴母细胞性淋巴瘤4例，急性髓系白血病15例，骨髓增生异常综合征2例；首次allo-HSCT供者类型包括无关供者12例、单倍体供者32例，所有患者在二次移植时均更换了供者；两次allo-HSCT间隔的中位时间为19.5（6～77）个月；二次移植前33例（75.0％）患者原发病为完全缓解，11例（25.0％）为未缓解；预处理方案包括全身放射治疗/氟达拉滨（38例）、白消安/氟达拉滨（4例）、全骨髓照射/氟达拉滨（1例）、白消安/克拉屈滨（1例）；采用环孢素A、霉酚酸酯、短程甲氨蝶呤及抗胸腺细胞球蛋白预防移植物抗宿主病（GVHD）。②所有患者均获得造血重建，Ⅱ～Ⅳ度、Ⅲ～Ⅳ度急性GVHD发生率分别为20.5％、9.1％，局限型、广泛型慢性GVHD发生率分别为20.5％、22.7％，巨细胞病毒、EB病毒感染发生率分别为29.5％、6.8％，出血性膀胱炎发生率为15.9％（均为Ⅰ～Ⅱ度）。③中位随访14（2～39）个月，移植后1年无病生存率、总生存率分别为72.5％（95％ *CI* 54.5％～84.3％）、80.6％（95％ *CI* 63.4％～90.3％），累积复发率为25.1％（95％ *CI* 13.7％～43.2％）。8例患者死亡，7例死于复发，1例死于感染，非复发死亡率为2.3％。④二次移植前完全缓解组、未缓解组移植后1年累积复发率分别为16.8％、48.1％（*P*＝0.026），无病生存率分别为79.9％、51.9％（*P*＝0.072）。⑤单因素分析显示二次移植前原发病是否完全缓解是预后的影响因素。

**结论:**

采用RIC方案、更换供者及移植后维持治疗等策略的二次移植治疗allo-HSCT后复发的恶性血液病具有较好的安全性及疗效。影响二次移植预后的最重要因素是二次移植前的疾病状态。

## Abstract (en)

**Objective:**

The purpose of this study was to assess the safety and efficacy of a second allogeneic hematopoietic stem cell transplantation（allo-HSCT）with reduced-intensity conditioning（RIC）in patients with hematological malignancies who had relapsed after the first allo-HSCT.

**Methods:**

Between April 2018 and June 2021, 44 patients with hematological malignancies（B-ALL 23, T-ALL/T-LBL 4, AML15, and MDS 2）were enrolled and retrospectively examined. Unrelated donors（*n*＝12）or haploidentical donors（*n*＝32）were used. Donors were replaced in all patients for the second allo-HSCT. Hematological and immunological germline predisposition genes and hematopoietic and immune function tests were used to select the best-related donor. Total body irradiation（TBI）/fludarabine（FLU）-based（*n*＝38）, busulfan（BU）/FLU-based（*n*＝4）, total marrow irradiation（TMI）/FLU-based（*n*＝1）, and BU/cladribine-based（*n*＝1）were the RIC regimens used. For graft versus host disease（GVHD）prevention, cyclosporine, mycophenolate mofetil, short-term methotrexate, and ATG were used. Eighteen（40.9％）of 44 patients with gene variations for which targeted medications are available underwent post-transplant maintenance therapy.

**Results:**

The median age was 25 years old (range: 7–55). The median interval between the first and second HSCT was 19.5 months (range: 6–77). Before the second allo-HSCT, 33 (75％) of the patients were in complete remission (CR), whereas 11 (25％) were not. All patients had long-term engraftment. The grade Ⅱ–Ⅳ GVHD and severe acute GVHD rates were 20.5％ and 9.1％, respectively. Chronic GVHD was found in 20.5％ of limited patterns and 22.7％ of severe patterns. CMV and EBV reactivation rates were 29.5％ and 6.8％, respectively. Hemorrhage cystitis occurred in 15.9％ of cases, grade Ⅰ or Ⅱ. The 1-yr disease-free survival (DFS), overall survival (OS), and cumulative recurrence incidence (RI) rates of all patients were 72.5％ (95％ *CI*, 54.5％–84.3％), 80.6％ (95％ *CI*, 63.4％–90.3％), and 25.1％ (95％ *CI*, 13.7％–43.2％), respectively, with a median follow-up of 14 (2–39) months. There were eight deaths (seven relapses and one infection). The rate of non-relapse mortality (NRM) was only 2.3％. The CR patients' 1-yr RI rate was significantly lower than the NR patients (16.8％ *vs* 48.1％, *P*＝0.026). The DFS rate in CR patients was greater than in NR patients, although there was no statistical difference (79.9％ *vs* 51.9％, *P*＝0.072). Univariate analysis revealed that CR before the second allo-HSCT was an important prognostic factor.

**Conclusion:**

With our RIC regimens, donor change, and post-transplant maintenance therapy, the second allo-HSCT in relapsed hematological malignancies after the first allo-HSCT is a safe and effective treatment with high OS and DFS and low NRM and relapse rate. The most important factor influencing the prognosis of the second allo-HSCT is the patient's illness condition before the transplant.

异基因造血干细胞移植（allo-HSCT）是恶性血液病的重要治疗手段，移植后疾病复发是导致治疗失败的最重要原因。移植后复发的患者预后差，可供选择的有效治疗手段不多[1]–[3]，二次allo-HSCT（二次移植）是可选方案之一。二次移植后1年、5年总生存（OS）率分别为40％、20％[4]，5年无病生存（DFS）率仅为15％[4]，10年OS率仅为（10±2）％[5]。近年来，我们采用减低强度预处理（RIC）、更换供者二次移植及移植后维持治疗策略治疗allo-HSCT后复发的恶性血液病患者，本研究对这些病例进行回顾性分析，以评估以上二次移植方案的疗效及安全性。

## 病例与方法

一、病例

本研究纳入2018年4月1日至2021年5月31日在北京高博博仁医院接受减低强度预处理（RIC）、更换供者二次移植的44例allo-HSCT后复发的恶性血液病患者，对其临床资料进行回顾性分析。

二、供者选择

首次移植的供者类型包括同胞相合供者5例、无关供者8例、单倍体供者30例、脐带血1例。二次移植包括无关供者12例（27.3％）、单倍体供者32例（72.7％）。本研究对29例（90.6％）选择单倍体供者的患者进行了血液和免疫系统疾病相关遗传易感基因的家系分析（外周血标本来自患者、父母及可能成为供者的兄弟姐妹及子女），并择优选择潜在的亲缘供者。32例单倍体供者造血及免疫功能检测（包括骨髓细胞形态学、染色体核型分析、淋巴细胞亚群、免疫球蛋白、NK细胞颗粒酶、穿孔素）均正常。

三、预处理方案

主要采用以全身放射治疗（TBI）或白消安（Bu）/氟达拉滨（Flu）为基础的RIC方案。TBI总量8 Gy或10 Gy，−9 d～−7 d或−8 d、−7 d；Bu 0.8 mg/kg每6 h 1次（体重<34 kg的儿童根据体重调整剂量），−9 d～−7 d；Flu 30 mg·m^−2^·d^−1^，−6 d～−2 d；阿糖胞苷（Ara-C）1～2 g·m^−2^·d^−1^，−6 d ～−4 d；司莫司汀（Me-CCNU）250 mg/m^2^，−3 d；兔抗人胸腺细胞免疫球蛋白总量5～6 mg/kg或抗人T细胞兔免疫球蛋白总量15 mg/kg，−5 d～−2 d。38例患者采用TBI/Flu方案（TBI 8 Gy 31例，10 Gy 7例），4例患者采用Bu/Flu方案，1例患者采用全骨髓照射（TMI，12 Gy，−8 d、−7 d）/Flu方案，1例患者采用Bu/克拉屈滨（5 mg/m^2^，−6 d～−2 d）方案。对于二次移植前原发病未缓解（NR）或微小残留病（MRD）阳性患者，预处理加用地西他滨（20 mg·m^−2^·d^−1^，−12 d～−10 d）或依托泊苷（200 mg·m^−2^·d^−1^，−11 d、−10 d）。

四、移植物抗宿主病（GVHD）和感染的预防

采用环孢素A或他克莫司、霉酚酸酯、短程甲氨蝶呤（MTX）方案预防GVHD。采用阿昔洛韦预防疱疹病毒感染，采用复方新诺明预防耶氏肺孢子菌肺炎，采用三唑类或棘白菌素类药物预防真菌感染。

五、维持治疗

44例患者中18例（40.9％）具有可供选择靶向药物的特定基因变异，二次移植后应用靶向药物进行维持治疗至移植后2年。针对NRAS、KRAS变异采用曲美替尼，针对TP53变异采用奥拉帕利、维奈克拉及地西他滨，针对IKZF1变异采用酪氨酸激酶抑制剂（TKI），针对BCR/ABL1融合基因采用敏感的TKI。

六、定义及标准

中性粒细胞植入标准：中性粒细胞计数>0.5×10^9^/L连续3 d。血小板植入标准：血小板计数>20×10^9^/L连续7 d且脱离血小板输注。急性GVHD（aGVHD）按照西奈山aGVHD国际联盟标准进行分度。慢性GVHD（cGVHD）采用西雅图工作组1980年提出的标准分为局限型和广泛型。OS时间定义为二次移植回输干细胞后至患者死亡或末次生存随访日。DFS时间定义为二次移植回输干细胞后至疾病复发或死亡。复发定义为获得CR后骨髓原始细胞再次≥5％，或外周血中又出现原始细胞，或出现髓外复发。非复发死亡定义为因非复发或非疾病进展原因造成的死亡。

七、随访

随访截止日期为2021年7月31日。通过查阅患者住院病历、门诊随访记录和电话随访获得患者生存资料。

八、统计学处理

采用SAS 9.4进行统计学分析。OS和DFS采用Kaplan-Meier分析方法，并进行Log-rank检验。复发率与非复发死亡率（NRM）进行竞争风险分析，采用Gray检验。以双侧*P*<0.05为差异具有统计学意义。

## 结果

一、临床特征

本研究纳入的44例患者中男21例，女23例，中位年龄25（7～55）岁。急性B淋巴细胞白血病（B-ALL）23例、急性T淋巴细胞白血病/T淋巴母细胞性淋巴瘤（T-ALL/T-LBL）4例、急性髓系白血病（AML）15例、骨髓增生异常综合征（MDS）2例。首次移植后骨髓复发32例（72.7％），髓外复发3例（6.8％），骨髓及髓外均复发9例（20.5％）。

移植前的疾病状态为完全缓解（CR）33例（75％），其中MRD阴性26例（59.1％），MRD阳性 7例（15.9％）；NR 11例（25％）。44例患者中以B-ALL为主（23例，占52.3％），其中14例二次移植前经CAR-T治疗后获得MRD阴性CR。两次移植间隔的中位时间为19.5（6～77）个月。二次移植采用与首次移植不同的预处理方案。二次移植中位单个核细胞（MNC）输注量为7.5（3.6～20.9）×10^8^/kg，中位CD34^+^细胞数输注量为4.5（1.9～9.7）×10^6^/kg。临床特征详见表1。

二、造血重建及移植后并发症

所有患者均获得成功、持久的造血重建，均达到完全供者嵌合。中性粒细胞中位植入时间为15（11～22）d。1例患者在移植后5个月死亡时血小板尚未植活，其余43例患者血小板植入的中位时间为15（10～240）d，移植后1、2个月血小板植入率分别为88.6％、95.4％。Ⅱ～Ⅳ度aGVHD发生率为20.5％，Ⅲ～Ⅳ度aGVHD发生率为9.1％。cGVHD发生率为43.2％（局限型20.5％，广泛型22.7％）。13例（29.5％）患者发生CMV激活，其中2例为CMV视网膜炎，其余为CMV血症。EBV激活3例（6.8％），2例为EBV血症，1例为EBV脑炎。出血性膀胱炎（HC）发生率为15.9％，其中Ⅰ度为13.6％，Ⅱ度为2.3％，无重度HC发生。移植相关血栓性微血管病（TA-TMA）的发生率为4.5％。

三、复发及生存

至随访截止时，二次移植后有10例患者复发，中位复发时间为119（32～650）d，移植前CR组（33例）、NR组（11例）各有5例患者复发。8例（18.2％）患者死亡，中位死亡时间为二次移植后165（79～780）d，其中7例死于复发，1例死于感染，NRM为2.3％。中位随访14（2～39）个月，二次移植后1年OS率、DFS率分别为80.6％（95％*CI* 63.4％～90.3％）、72.5％（95％*CI* 54.5％～84.3％）（图1），累积复发率（CIR）为25.1％（95％*CI* 13.7％～43.2％）。二次移植前CR组移植后1年OS率、DFS率、CIR分别为81.8％（95％*CI* 61.4％～92.0％）、79.9％（95％*CI* 60.5％～90.4％）、16.8％（95％*CI* 64.1％～92.7％），NR组移植后1年OS率、DFS率、CIR分别为76.2％（95％*CI* 33.2％～93.5％）、51.9％（95％*CI* 16.3％～79.0％）、48.1％（95％*CI* 16.3％～79.0％）。生存曲线见图2。

**表1:** 44例二次异基因造血干细胞移植治疗移植后复发恶性血液病患者的临床特征

指标	结果
性别［例（%）］	
男	21（47.7）
女	23（52.3）
二次移植时中位年龄［岁，*M*（范围）］	25（7~55）
二次移植时年龄	
≤14岁	9（20.5）
>14岁	35（79.5）
疾病类型［例（%）］	
B-ALL	23（52.3）
T-ALL/T-LBL	4（9.1）
AML	15（34.1）
MDS	2（4.6）
二次移植前疾病状态［例（%）］	
CR	33（75.0）
NR	11（25.0）
首次移植后缓解持续时间［例（%）］	
≤6个月	11（25.0）
>6个月	33（75.0）
首次移植后复发至二次移植时间［例（%）］	
≤5个月	24（54.5）
>5个月	20（45.5）
两次移植间隔时间［例（%）］	
≤12个月	12（27.3）
>12个月	32（72.7）
二次移植供者类型［例（%）］	
无关	12（27.3）
单倍体	32（72.7）
二次移植预处理方案［例（%）］	
TBI/Flu	38（86.4）
Bu/Flu	4（9.0）
TMI/Flu	1（2.3）
Bu/克拉屈滨	1（2.3）
二次移植造血干细胞来源［例（%）］	
BM+PBSC	28（63.6）
PBSC	16（36.4）

注 B-ALL：急性B淋巴细胞白血病；T-ALL：急性T淋巴细胞白血病；T-LBL：T淋巴母细胞性淋巴瘤；AML：急性髓系白血病；MDS：骨髓增生异常综合征；CR：完全缓解；NR：未缓解；TBI：全身放射治疗；Flu：氟达拉滨；Bu：白消安；TMI：全骨髓照射；BM：骨髓；PBSC：外周血干细胞

四、预后因素单因素分析

性别、二次移植时年龄是否大于14岁、首次allo-HSCT后CR时间是否>6个月、供者类型（无关供者/单倍体供者）、疾病种类（AML、MDS对B-ALL、T-ALL/T-LBL）、TBI剂量（8 Gy对10 Gy）对OS、DFS及CIR均无显著影响。两次移植间隔（>12个月对≤12个月）、首次移植后复发至二次移植的间隔（> 5个月对≤个5月）对CIR没有显著影响（*P*值分别为0.061、0.070）。二次移植前CR组、NR组CIR分别为16.8％、48.1％（*P*＝0.026），DFS率分别为79.9％、51.9％（*P*＝0.072）；OS率分别为81.8％、76.2％（*P*＝0.275）（表2）。

**图1:**
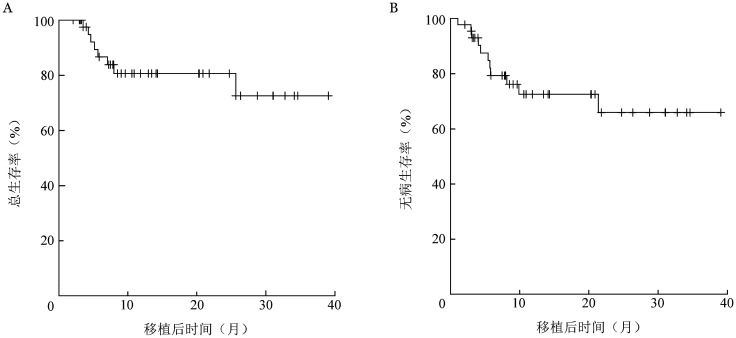
44例移植后复发恶性血液病患者二次移植后总生存（A）和无病生存（B）曲线

**图2:**
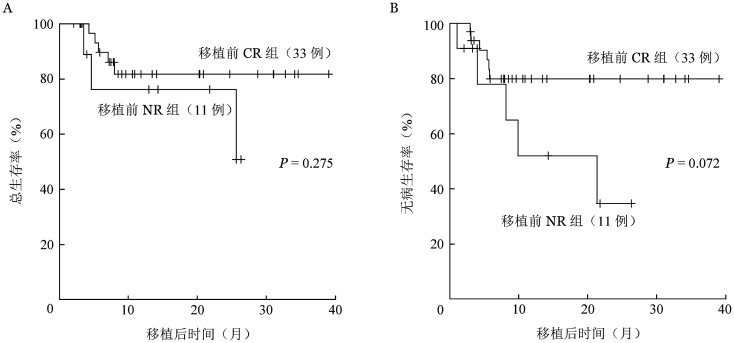
二次移植前疾病完全缓解（CR）和未缓解（NR）患者移植后总生存（A）及无病生存（B）曲线

**表2:** 44例异基因造血干细胞移植后复发恶性血液病患者二次移植主要结局影响因素的单因素分析

因素	例数	移植后1年总生存率（%）				移植后1年无病生存率（%）				移植后1年累积复发率（%）		
		值（95%*CI*）	*χ*^2^值	*P*值		值（95%*CI*）	*χ*^2^值	*P*值		值（95%*CI*）	*χ*^2^值	*P*值
性别			0.753	0.385			2.990	0.084			2.172	0.141
男	21	75.5（46.9~90.1）				58.2（31.1~77.8）				62.7（34.0~81.6）		
女	23	84.7（59.7~94.8）				85.6（61.5~95.1）				85.6（61.5~95.1）		
二次移植时年龄			0.291	0.590			<0.001	0.996			0.037	0.847
>14岁	35	83.5（64.8~92.8）				73.0（52.6~85.7）				76.0（55.5~88.0）		
≤14岁	9	68.6（21.3~91.2）				71.4（25.8~92.0）				71.4（25.8~92.0）		
首次移植缓解持续时间			0.435	0.510			0.670	0.413			1.208	0.272
>6个月	33	82.1（62.2~92.1）				73.4（51.3~86.6）				76.6（54.1~89.1）		
≤6个月	11	76.2（33.2~93.5）				68.2（29.7~88.6）				68.2（29.7~88.6）		
两次移植间隔			2.359	0.125			2.497	0.114			3.500	0.061
>12个月	32	85.1（65.1~94.2）				75.8（53.0~88.6）				79.1（55.8~91.0）		
≤12个月	12	68.6（30.5~88.7）				62.5（27.6~84.2）				62.5（27.6~84.2）		
首次移植后复发至二次移植时间			2.096	0.148			2.091	0.148			3.295	0.070
>5个月	20	86.5（55.8~96.5）				80.2（50.1~93.2）				85.6（53.3~96.2）		
≤5个月	24	75.9（51.4~89.2）				65.8（40.6~82.4）				65.8（40.6~82.4）		
二次移植供者类型			0.582	0.446			0.010	0.919			0.099	0.753
单倍体供者	32	81.5（61.0~91.9）				70.7（49.3~84.3）				73.7（52.1~86.8）		
无关供者	12	77.8（36.5~93.9）				77.8（36.5~93.9）				77.8（36.5~93.9）		
疾病诊断			0.232	0.630			0.027	0.869			0.067	0.795
AML、MDS	17	80.0（50.0~93.1）				71.7（40.1~88.6）				77.7（44.0~92.5）		
B-ALL、T-ALL/T-LBL	27	80.0（54.5~92.1）				72.4（47.9~86.8）				72.4（47.9~86.8）		
预处理TBI剂量			0.928	0.335			0.025	0.875			0.321	0.571
8 Gy	31	87.3（65.3~95.8）				74.6（51.1~88.0）				74.6（51.1~88.0）		
10 Gy	7	71.4（25.8~92.0）				71.4（25.8~92.0）				85.7（33.4~97.9）		
二次移植前疾病状态			1.190	0.275			3.232	0.072			4.930	0.026
CR	33	81.8（61.4~92.0）				79.9（60.5~90.4）				83.2（64.1~92.7）		
NR	11	76.2（33.2~93.5）				51.9（16.3~79.0）				51.9（16.3~79.0）		

注 AML：急性髓系白血病；B-ALL：急性B淋巴细胞白血病；T-ALL：急性T淋巴细胞白血病；T-LBL：T淋巴母细胞性淋巴瘤；MDS：骨髓增生异常综合征；TBI：全身放射治疗；CR：完全缓解；NR：未缓解

## 讨论

allo-HSCT后疾病复发是导致恶性血液病患者治疗失败的最常见的原因，二次移植可以使部分患者获得长期生存[5]–[8]。传统的清髓预处理方案（MAC）为TBI（12～14 Gy）/环磷酰胺（Cy）方案或Bu（16 mg/kg口服或12.8 mg/kg静脉输注）/Cy方案，然而二次移植采用MAC方案可导致NRM显著增加[9]，采用RIC方案可能是更好的选择[10]–[12]。来自欧洲血液及骨髓移植学会（EBMT）的资料显示，2 632例恶性血液病患者的研究显示二次移植后1年CIR为36％，NRM为33％[4]。Poon等[13]报道31例ALL患者二次移植后1年NRM为41％。国际血液和骨髓移植研究中心（CIBMTR）数据显示251例儿童及年轻成人急性白血病患者的研究显示CR组、NR组二次移植后1年CIR分别为29％、51％，NRM分别为25％、20％[12]。

本组allo-HSCT后复发的急性白血病及MDS患者采用RIC方案进行二次移植，TBI减量为总量8 Gy或10 Gy分次照射，Bu为0.8 mg/kg每6 h 1次×3 d静脉输注，用Flu取代Cy，86.4％（38/44）的患者采用TBI/Flu为主的方案。原发病以B-ALL为主（23例，占52.3％），其次为AML（15例，占34.1％），此外还有少数T-ALL/T-LBL（4例）和MDS（2例），其中11例（25％）二次移植前原发病为NR状态。所有患者均顺利获得持久植入，显示出良好的安全性及疗效。只有1例患者为非复发死亡，NRM仅为2.3％，显著低于既往的报告。二次移植后1年的复发率总体为25.1％，CR组为16.8％，生存得到明显改善。本研究采用RIC方案，尽管均为替代供者移植（无关或单倍体）且均应用了ATG，但aGVHD、cGVHD发生率及病毒激活率均较低，HC发生率仅为15.9％且无重度HC发生。

Yalniz等[14]采用二次移植治疗91例移植后复发的AML患者，移植后2年复发率为42％，73％的患者死于复发。来自CIBMTR的研究同样显示急性白血病复发为二次移植的主要死因（占53％）[12]。本研究共有8例患者死亡，其中7例死于复发，只有1例为非复发死亡。本组病例CIR较低，部分原因与移植前原发病获得CR及移植后维持治疗有关。

本研究所有患者二次移植时更换了供者。CIBMTR、EBMT数据及多个研究显示二次移植更换供者对OS、DFS或无白血病生存（LFS）、NRM、CIR等无影响[4],[9],[15]–[16]。个别研究结果提示二次移植选择同一供者是高LFS的有利因素[12]。我们选择更换供者是基于患者首次allo-HSCT后复发的部分原因与首次移植供者免疫细胞的移植物抗白血病/肿瘤效应减弱有关，更换免疫功能健康的供者有利于增加移植物抗白血病/肿瘤效应，尤其是对于存在人类HLA染色体杂合性缺失（HLA-loss）的患者。

在本研究中，我们对单倍体移植患者进行了血液和免疫系统疾病相关遗传易感基因的家系分析并择优选择供者，且所有供者造血与免疫功能检测均正常。通过上述遗传背景结合功能检查的方式排除了一些具有肿瘤易感基因及造血/免疫功能异常的亲缘供者，如果缺乏较理想的亲缘供者则优先选择无关供者。采用上述优选的供者进行二次移植将有利于建立强有力的造血及免疫系统，减少复发及感染的发生，从而提高二次移植的成功率。

以往研究显示，首次移植后缓解持续时间较长（>6个月、>10个月或>1年）、二次移植前CR、两次移植间隔较长（>430 d或>1年）、首次移植后复发到二次移植间隔≤5个月是OS、LFS或DFS的预后良好因素[5]–[7],[9],[12],[17]–[18]。本研究单因素分析显示，性别、二次移植时年龄是否大于14岁、首次移植后持续CR时间是否>6个月、移植类型（无关供者移植对单倍体移植）、疾病种类（AML、MDS对B-ALL、T-ALL/T-LBL）、两次移植间隔>12个月、首次移植后复发至二次移植间隔时间≤5个月对OS、DFS及CIR无显著影响，而二次移植前原发病CR组CIR显著低于NR组（16.8％对48.1％，*P*＝0.026），两组DFS与OS差异无统计学意义。

EBMT数据显示二次移植前原发病CR、使用TBI预处理方案是预后良好因素[5],[7]。本组病例获得较高的1年OS和DFS率可能与二次移植前大多数患者达到CR、且大多数患者采用TBI为主的预处理方案有一定关系。

本研究采用RIC方案、更换供者、新的供者选择模式及移植后的维持治疗等策略对移植后复发的急性白血病和MDS患者进行二次移植，显著降低了二次移植后aGVHD、cGVHD及病毒激活的发生率，NRM及CIR均显著减低，获得较好的疗效和安全性。本研究患者数量较少且随访时间较短，以上结论尚需进一步验证。

**利益冲突** 所有作者声明不存在利益冲突

**作者贡献声明** 赵永强：病例资料收集，数据分析，文章撰写；吴彤：研究设计及实施，数据分析，文章审核；其他作者：参与研究
